# Trastuzumab beyond progression in HER2‐positive metastatic breast cancer

**DOI:** 10.1111/tbj.14157

**Published:** 2021-01-20

**Authors:** Manwar Al‐Naqqash, Waleed Jasim Al‐Serarati, Tara Farooq Kareem

**Affiliations:** ^1^ Department of Surgery College of Medicine University of Baghdad Baghdad Iraq; ^2^ Department of Radiation Oncology Oncology Teaching Hospital Baghdad Medical City Complex Ministry of Health/Environment Baghdad Iraq; ^3^ Department of Oncology Baghdad Medical City Complex Ministry of Health and Environment Baghdad Iraq; ^4^ Department of Diagnostic Radiology Oncology Teaching Hospital Baghdad Medical City Complex Ministry of Health and Environment Baghdad Iraq

**Keywords:** breast cancer, HER2/neu, time to progression, trastuzumab

The data about continuing trastuzumab treatment beyond progression in HER2‐positive breast cancer are lacking in Iraq. This study aimed to investigate the use of trastuzumab in Iraqi women with metastatic HER2‐positive breast cancer.

A single‐arm noncomparative observational prospective study was conducted in Oncology Teaching Hospital between 2013 and 2019. The study included metastatic HER2‐positive breast cancer progressing after first‐line chemotherapy plus trastuzumab.

Only 16 (27%) cases had tumor progression. Median time to progression was 16 months (95% CI: 11.0‐20.9). 55 (93%) patients had a progression‐free survival of ≥6 months, whereas 32 (54%) had a progression‐free survival of ≥12 months.

The continuing trastuzumab beyond progression has clinical benefits in HER2‐positive metastatic breast cancer.

Worldwide, breast cancer is the most common cancer diagnosed among females^1^ and the leading cause of cancer‐related mortality.^2^ Approximately 66% of women died from BC live in low‐ and middle‐income countries.^3^ Treatment modalities of BC include surgery, radiotherapy, chemotherapy, hormonal therapy, targeted therapy, and immunotherapy. The clinical application of discovering HER2 in BC cells was the development of HER2‐targeted therapies.^4^

Trastuzumab was the first biological drug approved by the FDA in 1998 for the treatment of HER2‐positive BC.^5^ The addition of trastuzumab to chemotherapy in patients with metastatic BC had significantly increased overall response rate, duration of response, time to progression (TTP), and improved OS and DFS.^6^

The trastuzumab is the only anti‐HER2 agent approved in the country, and the objective was to investigate the use of trastuzumab in Iraqi women with metastatic HER2‐positive BC.

A single‐arm noncomparative observational prospective study was conducted in Oncology Teaching Hospital between 2013 and 2019. The study protocol was approved by the ethics committee of the hospital (MOH Approval No. 11). The data included age, weight, HER2 and hormone receptor status, metastasis sites, and disease stage, previous radiotherapy, adjuvant treatment, trastuzumab cycle number, and relapse‐free duration. The two study endpoints were BFS and TTP. The inclusion criteria were women with metastatic HER2‐positive BC and BC progressing after first‐line chemotherapy plus trastuzumab. We excluded those with a HER2‐negative and who stopped trastuzumab because of side effects.

Normality of continuous variables was tested by the Anderson‐Darling test. In case of normality, data were summarized using mean and standard deviation (SD); otherwise, median and interquartile range were used. Qualitative variables were described using frequency distribution. The Kaplan‐Meier analysis was used to estimate median time of the time to progression. IBM SPSS version 22.0.0 for Windows Release (Chicago, IL) and Prism version 7.00 for Windows (GraphPad Software) were used. *P*‐value was considered to be significant if less than .05.

The mean age was 51.4 ± 13.8 years. 28 (47.5%) patients had positive hormonal status, 13 (22%) patients underwent radiotherapy, and 56 (94.1%) patients received adjuvant chemotherapy. The most frequent metastatic site was lungs (n = 53; 89.9%) (Table 1).

**TABLE 1:** Baseline characteristics

Variables	Metastatic HER2 positive (N = 59)
Age (years), mean ± SD	51.4 ± 13.8
Weight (Kg), mean ± SD	75.5 ± 14.4
ER and/or PR positive, n (%)	28 (47.5)
Previous radiotherapy, n (%)	13 (22)
Adjuvant treatment, n (%)	56 (94.1)
Adjuvant trastuzumab cycles, mean ± SD	16.1 ± 6.9
Median relapse‐free interval, years (IQR)	12 (10‐24)

The median TTP (first progression) was 52 months (95% CI: 30.5‐73.4). After 2 years, 47 (79.6%) patients had a progression‐free period, and 32 (54.9%) patients had a 4‐year progression‐free period (Figure 1).

**FIGURE 1:**
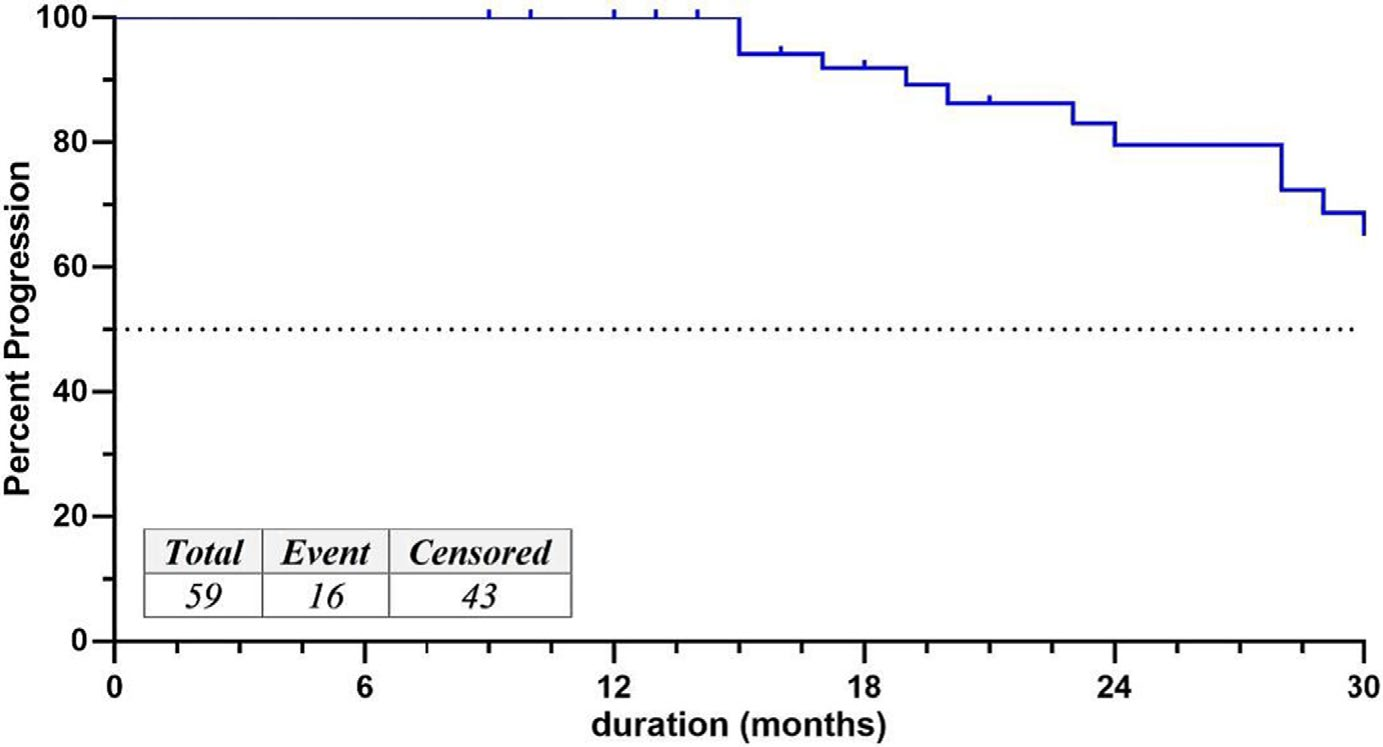
The Kaplan‐Meier curve for TTP since the first dose of first‐line trastuzumab in all patients [Color figure can be viewed at wileyonlinelibrary.com]

16/59 metastatic HER2‐positive cases had tumor progression after being treated with second‐line trastuzumab. Median TTP (primary endpoint) was 16 months (95% CI: 11‐20.9). 55/59 (93%) women had a PFS of ≥6 months, while 32 (54%) had a PFS of ≥12 months (Figure 2).

**FIGURE 2:**
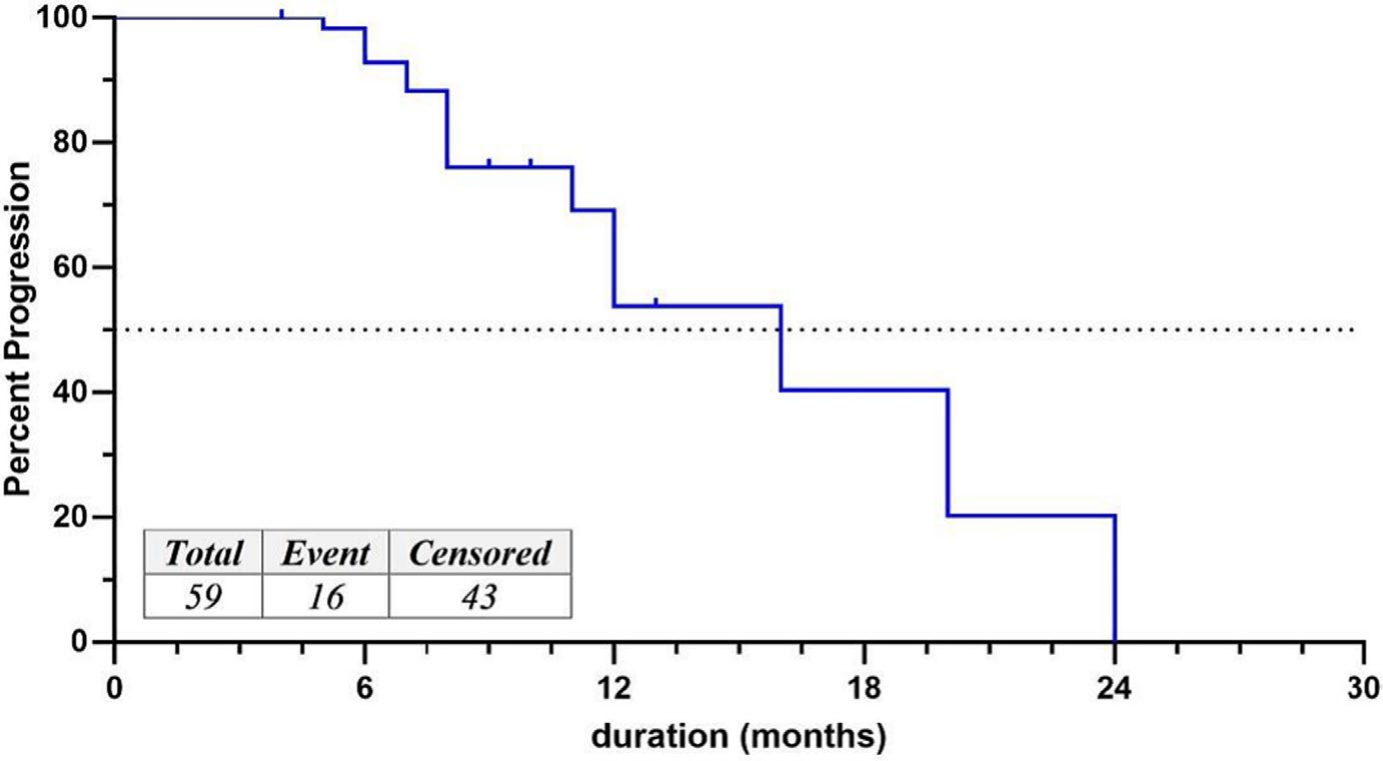
The Kaplan‐Meier curve for TTP after second‐line trastuzumab in all metastatic patients [Color figure can be viewed at wileyonlinelibrary.com]

The median TTP as primary endpoint was 16 months, with 27% of the patients having progressed at the end of the study. The median TTP was double what was reported in the GBG 26/BIG 03‐05 trial (median TTP of 8.2 months).^7^ The lower median TTP is related to the fact that eligible patients in the German study were only permitted to have one previous line of chemotherapy, while multiple lines were permitted in the current study.^7^ An Italian study found that the clinical outcome of patients who continued or discontinued trastuzumab is practically identical.^8^

Based on the US and Europe approvals, the duration of trastuzumab therapy is limited by the first evidence of disease progression.^9^ However, the off‐label use of trastuzumab beyond progression can be supported by certain mechanisms including direct antiproliferative activity, synergistic interaction with a number of standard chemotherapy agents, and antiangiogenic activity.^9^

The third ESO‐ESMO guidelines for advanced BC stated that data are sufficient to recommend continuing trastuzumab beyond progression, but the optimal duration and how many lines should it be used are currently unknown.^10^

To our knowledge, this is the first study in Iraq addressing the use of trastuzumab in BC beyond progression.

This study supports a growing body of evidence that is now consistently showing improved response rates and TTP when trastuzumab is continued beyond progression in HER2‐positive advanced BC.

## CONFLICT OF INTEREST

None.

## Data Availability

We make data available upon request at https://doi.org/10.5281/zenodo.4424435 (Manwar Alnaqqash (2021). Trastuzumab beyond progression in HER2‐positive metastatic breast cancer [data set]. Zenodo. http://doi.org/10.5281/zenodo.4424435). This project contains the following underlying data: OS.xlsx.
